# Inter- and Intra-Rater Reliability of Ultrasound Measurements of Superficial and Deep Fasciae Thickness in Upper Limb

**DOI:** 10.3390/diagnostics12092195

**Published:** 2022-09-09

**Authors:** Carmelo Pirri, Nina Pirri, Andrea Porzionato, Rafael Boscolo-Berto, Raffaele De Caro, Carla Stecco

**Affiliations:** 1 Department of Neurosciences, Institute of Human Anatomy, University of Padua, 35121 Padova, Italy; 2 Department of Medicine—DIMED, School of Radiology, Radiology Institute, University of Padua, 35128 Padova, Italy

**Keywords:** superficial fascia, deep fascia, ultrasonography, fascia, thickness, reliability

## Abstract

Ultrasound (US) imaging is increasingly the most used tool to measure the thickness of superficial and deep fasciae, but there are still some doubts about its reliability in this type of measurement. The current study sets out to assess the inter-rater and intra-rater reliability of US measurements of superficial and deep fasciae thicknesses in the arm and forearm. The study involved two raters: the first (R1) is an expert in skeletal–muscle US imaging and, in particular, the US assessment of fasciae; the second (R2) is a radiologist resident with 1 year’s experience in skeletal–muscle US imaging. R2, not having specific competence in the US imaging of fasciae, was trained by R1. R1 took US images following the protocol by Pirri et al. 2021, and the US-recorded images were analyzed separately by the two raters in different sessions. Each rater measured both types of fasciae at different regions and levels of the arm and forearm. Intra- and inter-rater reliability was excellent for the deep fascia and good and excellent for the superficial fascia according to the different regions/levels (for example for the anterior region of the arm: deep fascia: Ant 1: ICC_2,2_ = 0.95; 95% CI = 0.81–0.98; superficial fascia: Ant 1: ICC_2,2_ = 0.85, 95% CI = 0.79–0.88). These findings confirm that US imaging is a reliable and cost-effective tool for evaluating both fasciae, superficial and deep.

## 1. Introduction

Different imaging techniques, such as magnetic resonance imaging (MRI) [1,2] and computerized tomography scanning (CTS) [3], are used to measure different parameters to assess musculoskeletal structures.

Being more expensive, these methods are considered unsuitable for managing some pathological conditions and not practical for large-scale studies. Nowadays, musculoskeletal ultrasound (US) imaging, being a readily accessible, cost-effective, and reliable imaging method, has increased its field of action in rehabilitation medicine [4]. Playing an important role as pain generators in numerous musculoskeletal injuries, the thickness of various anatomical structures such as tendons, ligaments, and muscles are measured and monitored [5,6,7,8]. Moreover, knowing the size and thickness of these structures is important for specialists managing patients with different musculoskeletal injuries [7,8,9,10,11,12].

The reliability of US imaging for measuring these structures has also been assessed in various other studies [7,8,9,10,13]. While there have been numerous investigations on the different musculoskeletal structures, fascial layers have not been studied in the upper limb except in two studies about the thickness of deep and superficial fascia in the arm and forearm [14,15]. The thickness of tendons, ligaments, and muscles has been reported, but the complex system of the fascia that covers the muscles and organizes the subcutaneous tissue has not been assessed.

We know from histological studies [16] that the fasciae of the upper limb are composed of superficial fascia inside the subcutaneous tissue and deep fascia that envelop the muscles, as in other topographical regions [14,15,17]. Numerous studies have reported the crucial role of deep fascia thickness knowledge in the management of regional anesthesiology for fascia plane, inter-fascial, and nerve blocks, reducing the risk of nerve damage [14,18]. Other studies have confirmed the importance of deep fascia thickness in the acute compartment syndrome in the upper limb [14,19]. Moreover, many studies in recent years have highlighted in plastic and reconstructive surgery how knowing the exact thickness of a patient’s superficial fascia and predicting the seal of it will reduce damage while making a flap [14,15,20].

Some studies investigating the value of the US assessment of fasciae in other areas have demonstrated the efficacious evaluation of deep fascia thickness [17,21] and measurements of the thickness of superficial fascia and of subcutaneous tissue at a lower cost than other non-invasive methods [22,23]. Furthermore, although US imaging appears to be the best type of imaging for studying and assessing fasciae, standard reference values and their appearance on scans have not been entirely clarified. To date, some studies have examined different types of fasciae by US imaging, reporting reliable results in US fasciae thickness assessments [17,21,22,23]. Pirri et al. [23] demonstrated that expertise in US imaging and expertise in identifying anatomical landmarks from a fascial point of view are fundamental for reliable results, pointing out possible sources of error that lie in measurements or interpretation errors linked to the US technique, including difficulty in identifying anatomical landmarks during the US exam [23]. In addition, a known obstacle in ultrasound imaging is obtaining exact US images. Nevertheless, despite rigorous efforts and extensive previous training, there may be measurement inaccuracies by distortions that could influence the measurements, as demonstrated for other anatomical structures [24]. Furthermore, the experience level of the raters, especially in these types of assessments, must be considered a possible cause of error in the evaluation of fasciae thickness [23]. Finally, in CT and MRI, the measurements are based on an identical data set, whereas in US imaging, the measurements of the different raters are based on different images that they acquire themselves. However, only a few studies have focused on inter-rater reliability [23,25] between raters with different levels of experience in fascial anatomy.

The current study sets out to assess the inter-reliability of US thickness measurements of superficial and deep fasciae in the upper limb of healthy volunteers between raters with different levels of experience in the sono-anatomy of fasciae (an expert and a novice), performing the evaluations at different times on the same US images taken by the expert rater to eliminate error in the acquisition of the images.

## 2. Materials and Methods

### 2.1. Study Design

A cross-sectional study based on the Strengthening Reporting of Observational Study in Epidemiology (STROBE) statement was conducted [26] in order to assess intra- and inter-reliability in the measurements of the US thicknesses of superficial fascia and deep fascia of the arm and forearm in different regions and levels of the arm and the forearm. The Helsinki Declaration and human experimentation rules [27] were considered, and the Ethics Committee of the University of Padua evaluated the research. All participants were informed prior to inclusion in the project and provided a written consent form.

### 2.2. Participants

A total sample of 30 subjects was recruited, aged between 20 and 60 years. The participants were excluded if they had any upper extremity injuries (e.g., previous fractures, tendinopathies, tendon ruptures, or neuropathy injuries; past diagnosis of a neuromusculoskeletal condition of the arm or forearm, e.g., use of palmar orthoses, carpal tunnel syndrome, etc.; or past diagnosis of a neuro-musculoskeletal condition of the arm or forearm, e.g., degeneration or inflammation of the homerus periosteum) or surgeries; had severe orthopedic, neuronal, psychiatric, cardiopulmonary, or endocrine diseases; were under 18 years old or pregnant; had a chronic skin condition (eczema, psoriasis, lymphedema, lipedema, etc.); had previous severe trauma in the inferior limbs, collagen disorder (scleroderma, mixed connective tissue disorder, etc.), and/or a chronic medical condition requiring the intake of medications. The enrolment of the subjects was performed by a specialized medical doctor with more than 5 years of experience in physical and rehabilitation medicine. For transparency, the studied group was the same group of subjects as in a previous paper by the authors [15] that had different research objectives.

### 2.3. Raters

Two physicians carried out the US image assessments: the first rater (Rater 1 = R1) is a specialist in physical and rehabilitation medicine and an expert with 7 years’ experience in skeletal–muscle US imaging and, in particular, in the US assessment of fasciae; the second rater (Rater 2 = R2) is a radiologist resident with 1 year’s experience in skeletal–muscle US imaging. R2, not having specific competence in the US imaging of fasciae, was trained by R1. The training program consisted of an initial 5 h instruction session, followed by 60 h of vision/hands-on software experience over a period of 3 months to visualize the structures and measure the fasciae.

### 2.4. Ultrasonography Imaging Measurements

**Figure 1:**
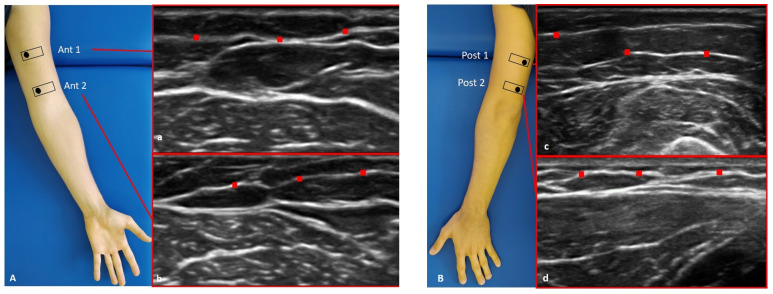

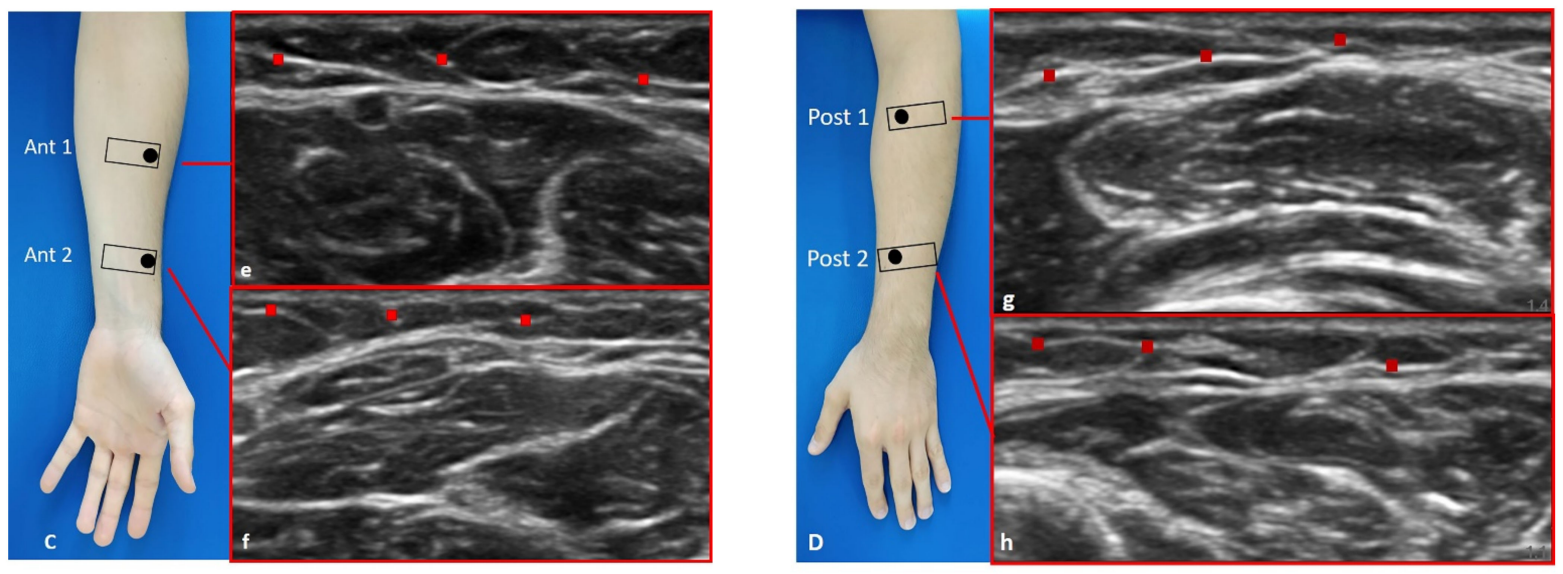
Ultrasound (US) images of the superficial fascia of: the anterior region of the arm (**A**) and of the forearm (**C**); the posterior region of the arm (**B**) and of the forearm (**D**). Anterior regions (**A**,**C**) at levels Ant 1 (**a**,**e**) and Ant 2 (**b**,**f**). Posterior regions (**B**,**D**) at levels Post 1 (**c**,**g**) and Post 2 (**d**,**h**). Probe: black rectangle. Red dashes: superficial fascia. Reprinted with permission from [15].

**Figure 2:**
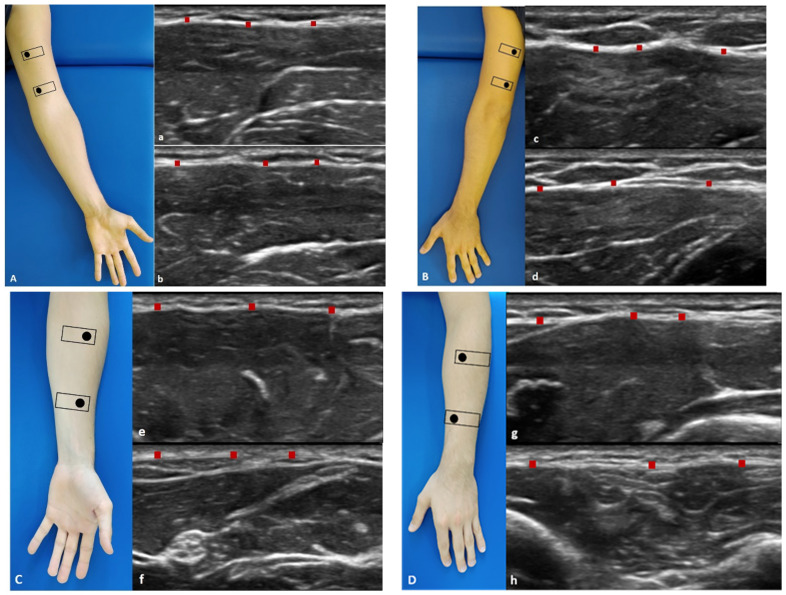
Ultrasound (US) images of the deep fascia of: the anterior region of the arm (**A**) and of the forearm (**C**); the posterior region of the arm (**B**) and of the forearm (**D**). Anterior region (**A**,**C**) at the levels Ant 1 (**a**,**e**) and Ant 2 (**b**,**f**). Posterior region (**B**,**D**) at levels Post 1 (**c**,**g**) and Post 2 (**d**,**h**). Probe: black rectangle. Red dashes: deep fascia. Reprinted with permission from [14].

US images of the superficial and deep fasciae were obtained using a high-resolution device (Sonosite Edge II, FUJIFILM, Inc. 21919, Bothell, WA, USA) with a 6–15 MHz linear transducer (HLF50x, Sonosite Edge II, FUJIFILM, Inc. 21919, Bothell, WA, USA) and a screen resolution of 1680 × 1050 pixels. Images were taken of the arm and forearm with a specific protocol in accordance with Pirri et al. [14,15] (Figure 1 and Figure 2).

The US images were frozen, captured, and acquired at the end of each assessment by R1; the superficial and deep fascia thicknesses were measured independently using Image J software (available online: https://imagej.nih.gov/ij/) by the two raters (R1 and R2). To eliminate the influence of possible thickness variations, three equidistant regions of interest per image/level for superficial fascia and deep fascia were measured; in each of them, three points representing the best visibility for each superficial fascia and deep fascia layer were measured, and the resulting values were averaged for analysis. The raters followed the same protocol to ensure that each point of superficial and deep fascia in the arm and in the forearm was quantified in the same way. Moreover, the same procedure of image assessment was repeated twice at different times (after seven days, the protocol design of image assessment was repeated identically to the first session of measure) to calculate the intra- and inter-reliability of the measurements.

### 2.5. Statistical Analysis

Statistical analysis was carried out with SPSS version 21 (SPSS Inc., Chicago, IL, USA). Data were expressed as mean ± standard deviation (SD). The Kolmogorov–Smirnov test was used to assess normality. The intra- and inter-reliability of the US superficial and deep fasciae thicknesses were quantified using the two-way mixed model intra-class correlation coefficient (ICC)—respectively, ICC_3,2_ for intra-reliability and ICC_2,2_ for inter-reliability. The ICC values were interpreted as poor when below 0.5, as moderate when between 0.5 and 0.75, as good when between 0.75 and 0.90, and as excellent when above 0.90 [28]. The 95% limits of agreement (LoAs) between sessions and raters express the degree of error proportional to the mean of the measurement units; these statistics were calculated using methods described by Bland and Altman [29]. Their plots were used to show the difference between each pair of measurements on the *y*-axis against the mean of each pair of measurements on the *x*-axis. Bland–Altman plots were drawn to graphically assess the magnitude of the differences (bias) between measurements and raters. If the differences between the measurements tend to agree, the results will be close to zero. In particular, the 95% CI of the mean difference illustrates the magnitude of the systematic difference. A *p* < 0.05 was always considered the limit for statistical significance. Finally, differences between the measurements of the raters were analyzed by Student’s *t*-test for paired samples.

## 3. Results

### 3.1. Descriptive Data 

A total of 30 subjects (16 females and 14 males) participated in this study. The descriptive data of the sample are summarized in Table 1.

### 3.2. Intra-Rater Reliability of Deep Fascia of Arm

#### 3.2.1. Rater 1

**Figure 3:**
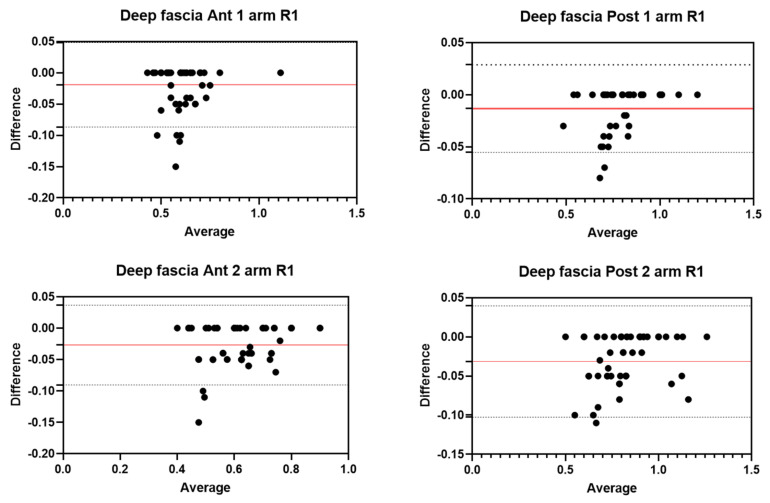
Bland–Altman plot comparing two sessions of Rater 1 (R1) measuring the US thickness of the deep fascia of the arm for the different levels. Red line = bias or mean difference; dashed lines: 95% limits of agreement (LoAs).

Analysis of the reliability of the US measurements of the thickness of the deep fascia of the arm between the first session and the second session by R1 (Table 2) showed excellent intra-rater values for the Ant 1 level (ICC_3,2_ = 0.97; 95% CI = 0.91–0.98) and the Ant 2 level (ICC_3,2_ = 0.97; 95% CI = 0.84–0.98). Moreover, excellent intra-rater values were also confirmed for the posterior region at the Post 1 level (ICC_3,2_ = 0.99; 95% CI = 0.97–0.99) and the Post 2 level (ICC_3,2_ = 0.97; 95% CI = 0.87–0.99). There were no statistical differences between the first and the second sessions (*p >* 0.05). The bias, SD of bias, 95% CI, and 95% LoA were, respectively, for the different regions/levels: Ant 1: −0.02, 0.03, −0.01 to −0.01, −0.08–0.04; Ant 2: −0.03, 0.03, −0.02 to −0.02, −0.09–0.03; Post 1: −0.01, 0.02, −0.01 to −0.01, −0.05–0.02; Post 2: −0.03, 0.04, −0.04 to −0.02, −0.10–0.03. Figure 3 shows the Bland–Altman plots for the US measurements of the thickness of the deep fascia of the arm between the first session and the second session.

#### 3.2.2. Rater 2

**Figure 4:**
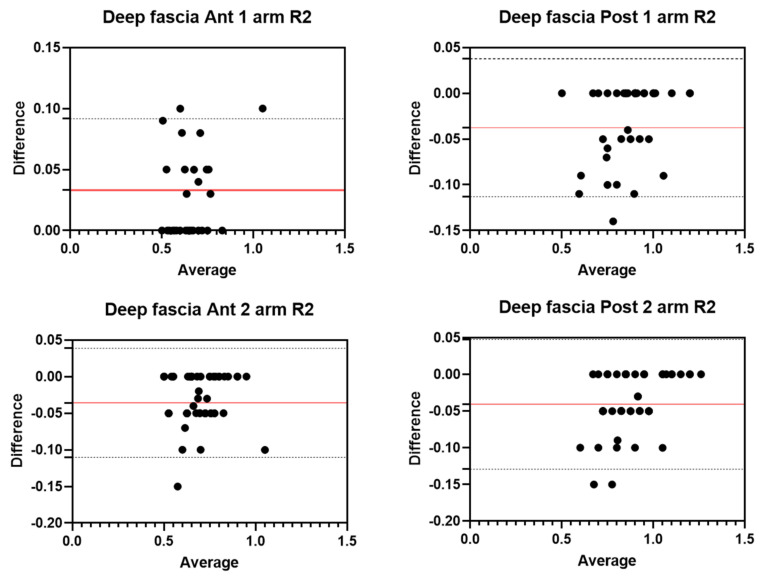
Bland–Altman plot comparing two sessions of Rater 2 (R2) measuring the US thickness of the deep fascia of the arm for the different levels. Red line = bias or mean difference; dashed lines: 95% limits of agreement (LoAs).

Analysis of the reliability of the US measurements of the thickness of the deep fascia of the arm between the first session and the second session by R2 (Table 3) showed excellent intra-rater values for the Ant 1 level (ICC_3,2_ = 0.94; 95% CI = 0.53–0.98) and the Ant 2 level (ICC_3,2_ = 0.95; 95% CI = 0.69–0.98). Moreover, excellent intra-rater values were also confirmed for the posterior region at the Post 1 level (ICC_3,2_ = 0.95; 95% CI = 0.71–0.98) and the Post 2 level (ICC_3,2_ = 0.96; 95% CI = 0.77–0.98). There were no statistical differences between the first session and the second session (*p* > 0.05). The bias, SD of bias, 95% CI, and 95% LoA were, respectively, for the different regions/levels: Ant 1: 0.03, 0.03, 0.02 to 0.04, −0.02–0.09; Ant 2: −0.04, 0.04, −0.05 to −0.03, −0.11–0.03; Post 1: −0.04, 0.04, −0.05 to −0.03, −0.11–0.03; Post 2: −0.04, 0.05, −0.05 to −0.03, −0.12–0.04. Figure 4 shows the Bland–Altman plots for the US measurements of the thickness of the deep fascia of the arm between the first session and the second session.

### 3.3. Intra-Rater Reliability of Deep Fascia of Forearm

#### 3.3.1. Rater 1

**Figure 5:**
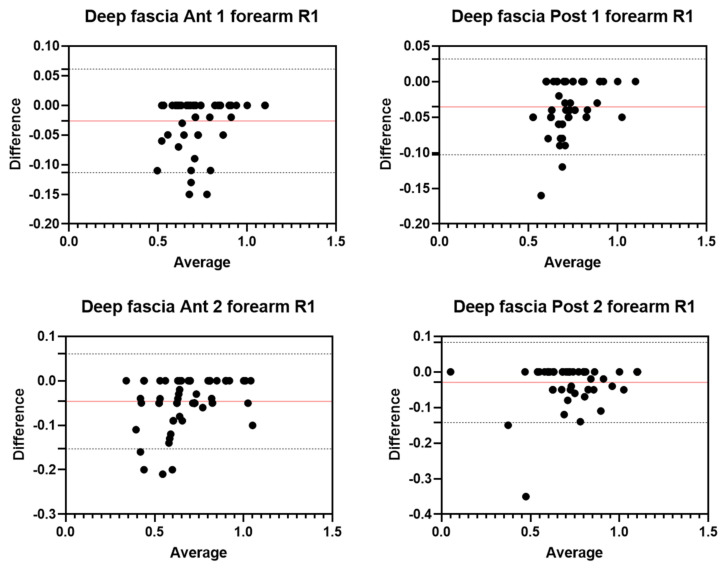
Bland–Altman plot comparing two sessions of Rater 1 (R1) measuring the US thickness of the deep fascia of the forearm for the different levels. Red line = bias or mean difference; dashed lines: 95% limits of agreement (LoAs).

Analysis of the reliability of the US measurements of the thickness of the deep fascia of the forearm between the first session and the second session by R1 (Table 4) showed excellent intra-rater values for the Ant 1 level (ICC_3,2_ = 0.96; 95% CI = 0.90–0.98) and the Ant 2 level (ICC_3,2_ = 0.95; 95% CI = 0.80–0.98). Moreover, excellent intra-rater values were also confirmed for the posterior region at the Post 1 level (ICC_3,2_ = 0.95; 95% CI = 0.65–0.98) and the Post 2 level (ICC_3,2_ = 0.97; 95% CI = 0.93–0.98). There were no statistical differences between the first session and the second session (*p >* 0.05). The bias, SD of bias, 95% CI, and 95% LoA were, respectively, for the different regions/levels: Ant 1: −0.03, 0.04,−0.03 to −0.01, −0.11–0.06; Ant 2: −0.05, 0.05, −0.06 to −0.03, −0.15–0.06; Post 1: −0.04, 0.03, −0.04 to −0.03, −0.1–0.03; Post 2: −0.03, 0.1, −0.04 to −0.1, −0.14–0.08. Figure 5 shows the Bland–Altman plots for the US measurements of the thickness of the deep fascia of the arm between the first session and the second session.

#### 3.3.2. Rater 2

**Figure 6:**
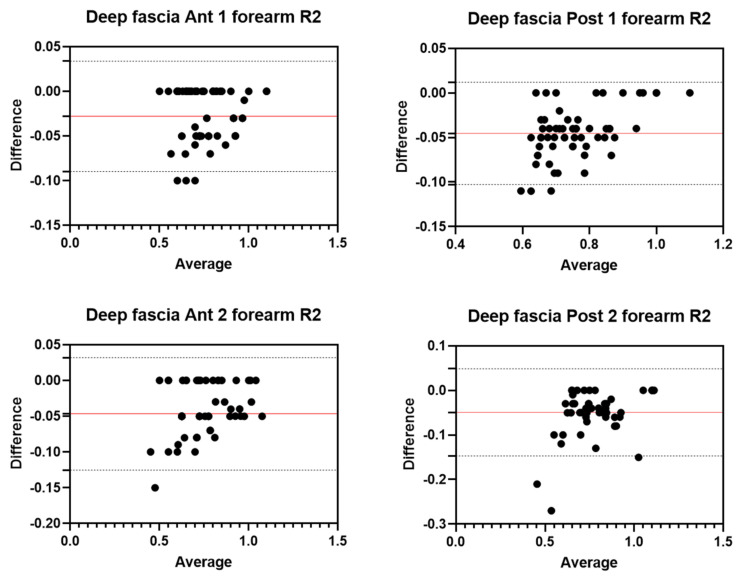
Bland–Altman plot comparing two sessions of Rater 2 (R2) measuring the US thickness of the deep fascia of the forearm for the different/levels. Red line = bias or mean difference; dashed lines: 95% limits of agreement (LoAs).

Analysis of the reliability of the US measurements of the thickness of the deep fascia of the forearm between the first session and the second session by R1 (Table 5) showed excellent intra-rater values for the Ant 1 level (ICC_3,2_ = 0.97; 95% CI = 0.86–0.99) and the Ant 2 level (ICC_3,2_ = 0.96; 95% CI = 0.62–0.98). Moreover, excellent intra-rater values were also confirmed for the posterior region at the Post 1 level (ICC_3,2_ = 0.94; 95% CI = 0.67–0.98) and the Post 2 level (ICC_3,2_ = 0.95; 95% CI = 0.67–0.98). There were no statistical differences between the first session and the second session (*p >* 0.05). The bias, SD of bias, 95% CI, and 95% LoA were, respectively, for the different regions/levels: Ant 1: −0.03, 0.03, −0.04 to −0.02, −0.08–0.03; Ant 2: −0.05, 0.04, −0.06 to −0.04, −0.12–0.03; Post 1: −0.05, 0.03, −0.05 to −0.04, −0.10–0.01; Post 2: −0.05, 0.05, −0.06 to −0.04, −0.14–0.04. Figure 6 shows the Bland–Altman plots for the US measurements of the thickness of the deep fascia of the arm between the first session and the second session.

### 3.4. Intra-Rater Reliability of Superficial Fascia of Arm

#### 3.4.1. Rater 1

**Figure 7:**
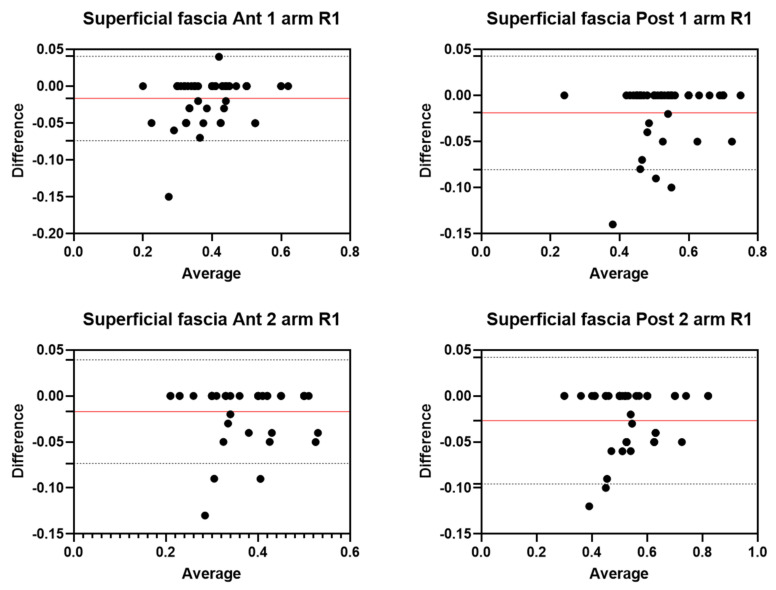
Bland–Altman plot comparing two sessions of Rater 1 (R1) measuring the US thickness of the superficial fascia of the arm for the different levels. Red line = bias or mean difference; dashed lines: 95% limits of agreement (LoAs).

Analysis of the reliability of the US measurements of the thickness of the superficial fascia of the arm between the first session and the second session by R1 (Table 6) showed excellent intra-rater values for the Ant 1 level (ICC_3,2_ = 0.96; 95% CI = 0.91–0.98) and the Ant 2 level (ICC_3,2_ = 0.96; 95% CI = 0.90–0.98). Moreover, excellent intra-rater values were also confirmed for the posterior region at the Post 1 level (ICC_3,2_ = 0.96; 95% CI = 0.90–0.98) and the Post 2 level (ICC_3,2_ = 0.96; 95% CI = 0.84–0.98). There were no statistical differences between the first session and the second session (*p >* 0.05). The bias, SD of bias, 95% CI, and 95% LoA were, respectively, for the different regions/levels: Ant 1: −0.02, 0.03, −0.03 to −0.01, −0.07–0.04; Ant 2: −0.02, 0.03, −0.02 to −0.01, −0.07–0.03; Post 1: −0.02, 0.03, −0.03 to −001, −0.08–0.04; Post 2: −0.03, 0.04, −0.04 to −0.02, −0.09–0.04. Figure 7 shows the Bland–Altman plots for the US measurements of the thickness of the deep fascia of the arm between the first session and the second session.

#### 3.4.2. Rater 2

**Figure 8:**
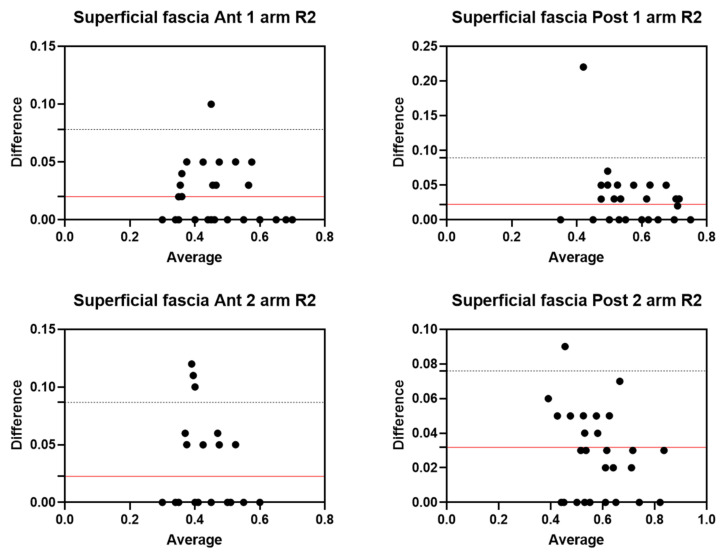
Bland–Altman plot comparing two sessions of Rater 2 (R2) measuring the US thickness of the superficial fascia of the arm for the different/levels. Red line = bias or mean difference; dashed lines: 95% limits of agreement (LoAs).

Analysis of the reliability of the US measurements of the thickness of the superficial fascia of the arm between the first session and the second session by R2 (Table 7) showed excellent intra-rater values for the Ant 1 level (ICC_3,2_ = 0.96; 95% CI = 0.87–0.98) and the Ant 2 level (ICC_3,2_ = 0.93; 95% CI = 0.78–0.97). Moreover, excellent intra-rater values were also confirmed for the posterior region at the Post 1 level (ICC_3,2_ = 0.94; 95% CI = 0.84–0.97) and the Post 2 level (ICC_3,2_ = 0.95; 95% CI = 0.82–0.98). There were no statistical differences between the first session and the second session (*p >* 0.05). The bias, SD of bias, 95% CI, and 95% LoA were, respectively, for the different regions/levels: Ant 1: 0.02, 0.03, −0.04 to −0.02, −0.03–0.07; Ant 2: 0.02, 0.03, 0.01 to 0.03, −0.04–0.08; Post 1: 0.02, 0.03, 0.01 to 0.03, −0.04–0.08; Post 2: 0.03, 0.02, 0.03 to 0.04, −0.01–0.07. Figure 8 shows the Bland–Altman plots for the US measurements of the thickness of the deep fascia of the arm between the first session and the second session.

### 3.5. Intra-Rater Reliability of Superficial Fascia of Forearm

#### 3.5.1. Rater 1

**Figure 9:**
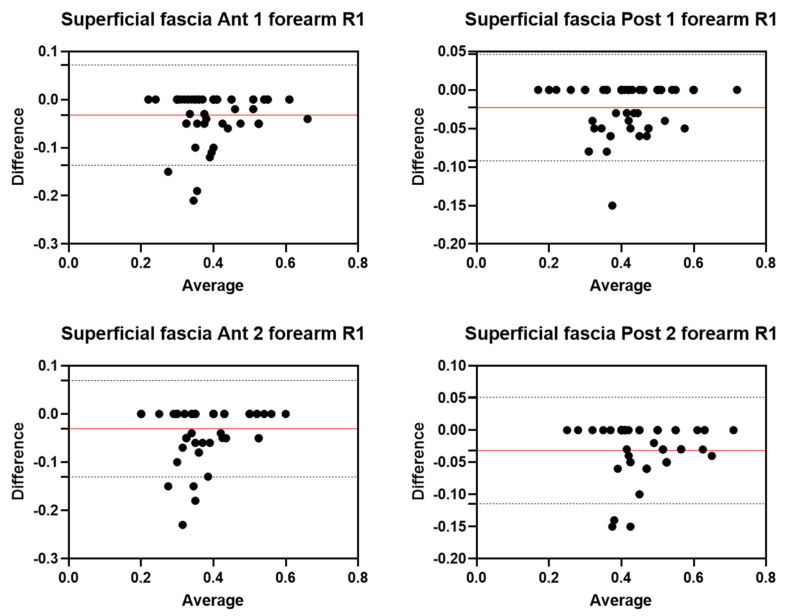
Bland–Altman plot comparing two sessions of Rater 1 (R1) measuring the US thickness of the superficial fascia of the forearm for the different levels. Red line = bias or mean difference; dashed lines: 95% limits of agreement (LoAs).

Analysis of the reliability of the US measurements of the thickness of the superficial fascia of the forearm between the first session and the second session by R1 (Table 8) showed excellent intra-rater values for the Ant 1 level (ICC_3,2_ = 0.90; 95% CI = 0.75–0.95) and the Ant 2 level (ICC_3,2_ = 0.89; 95% CI = 0.90–0.98). Moreover, excellent intra-rater values were also confirmed for the posterior region at the Post 1 level (ICC_3,2_ = 0.96; 95% CI = 0.89–0.98) and the Post 2 level (ICC_3,2_ = 0.91; 95% CI = 0.70–0.96). There were no statistical differences between the first session and the second session (*p >* 0.05). The bias, SD of bias, 95% CI, and 95% LoA were, respectively, for the different regions/levels: Ant 1: −0.03, 0.05, −0.05 to −0.02, −0.13–0.07; Ant 2: −0.03, 0.05, −0.04 to −0.02, −0.13–0.06; Post 1: −0.02, 0.04, −0.03 to −0.01, −0.09–0.04; Post 2: −0.03, 0.04, −0.04 to −0.02, −0.11–0.05. Figure 9 shows the Bland–Altman plots for the US measurements of the thickness of the deep fascia of the arm between the first session and the second session.

#### 3.5.2. Rater 2

**Figure 10:**
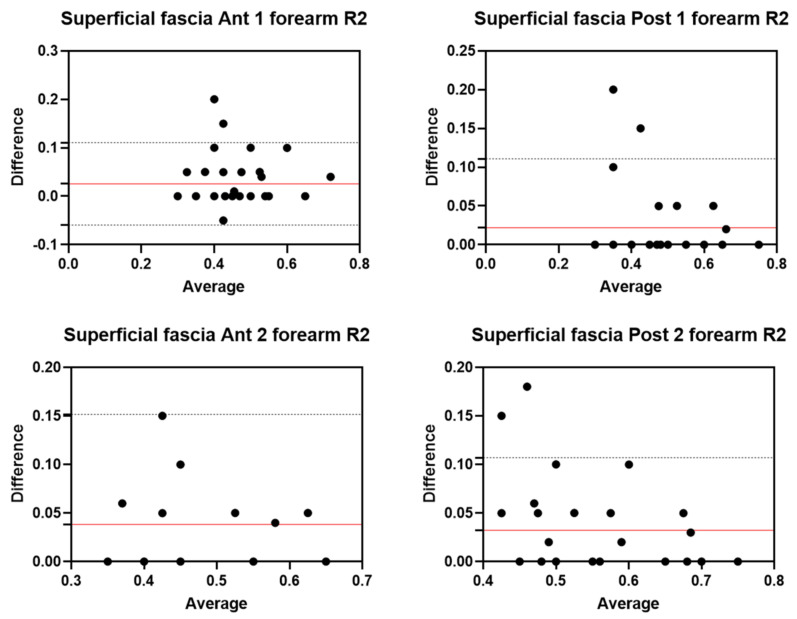
Bland–Altman plot comparing two sessions of Rater 2 (R2) measuring the US thickness of the superficial fascia of the forearm for the different levels. Red line = bias or mean difference; dashed lines: 95% limits of agreement (LoAs).

Analysis of the reliability of the US measurements of the thickness of the superficial fascia of the forearm between the first session and the second session by R1 (Table 9) showed excellent intra-rater values for the Ant 1 level (ICC_3,2_ = 0.90; 95% CI = 0.76–0.95) and good values for the Ant 2 level (ICC_3,2_ = 0.78; 95% CI = 0.55–0.89). Moreover, excellent intra-rater values were also confirmed for the posterior region at the Post 1 level (ICC_3,2_ = 0.92; 95% CI = 0.84–0.96) and the Post 2 level (ICC_3,2_ = 0.89; 95% CI = 0.57–0.95). There were no statistical differences between the first session and the second session (*p >* 0.05). The bias, SD of bias, 95% CI, and 95% LoA were, respectively, for the different regions/levels: Ant 1: 0.03, 0.04, 0.02 to 0.04, −0.05–0.11; Ant 2: 0.04, 0.06, 0.02 to 0.03, −0.07–0.15; Post 1: 0.02, 0.05, 0.02 to 0.04, −0.06–0.11; Post 2: 0.03, 0.04, −0.04–0.1. Figure 10 shows the Bland–Altman plots for the US measurements of the thickness of the deep fascia of the arm between the first session and the second session.

### 3.6. Inter-Rater Reliability of Deep Fascia

**Figure 11:**
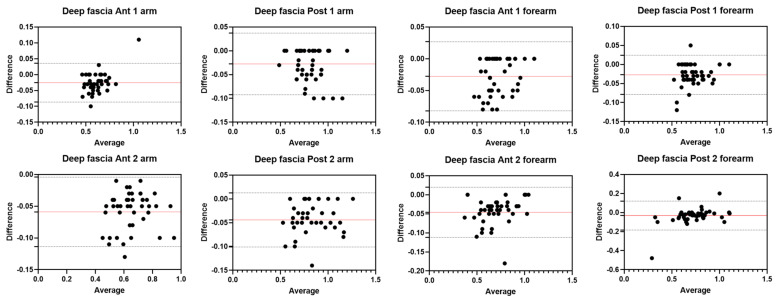
Bland–Altman plot comparing two raters (R1 and R2) measuring the US thicknesses of the deep fascia of the arm and forearm for the different levels. Red line = bias or mean difference; dashed lines: 95% limits of agreement (LoAs).

Analysis of the reliability of the US measurements of the thickness of the deep fascia of the arm and forearm between the two raters (R1 and R2) showed excellent inter-rater reliability for all regions/levels for both arm and forearm (Table 10). Bland–Altman plots comparing the two raters for the different topographical regions/levels of deep fascia are shown in Figure 9. There were no statistical differences between the first session and the second session (*p >* 0.05). The bias, SD of bias, 95% CI, and 95% LoA were, respectively, for the different regions/levels of the arm: Ant 1: −0.03, 0.03, −0.03 to −0.02, −0.08–0.03; Ant 2: −0.05, 0.03, −0.1 to −0.05, −0.11–0.004; Post 1: −0.03, 0.03, −0.04 to −0.02, −0.09–0.03; Post 2: −0.04, 0.03, −0.05 to −0.04, −0.10–0.01. The bias, SD of bias, 95% CI, and 95% LoA were, respectively, for the different regions/levels of the forearm: Ant 1: −0.03, 0.03,−0.03 to −0.02, −0.08–0.02; Ant 2: −0.05, 0.03, −0.05 to −0.04, −0.11–0.01; Post 1: −0.03, 0.03, −0.03 to 0.02, −0.07–0.02; Post 2: −0.03, 0.08, −0.05 to −0.01, −0.18–0.12. Figure 11 shows the Bland–Altman plots for US measurements of the thickness of the deep fascia of the arm between the first session and the second session.

### 3.7. Inter-Rater Reliability Superficial Fascia

**Figure 12:**
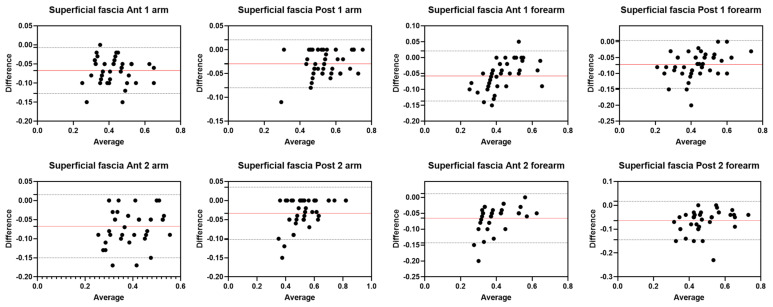
Bland–Altman plot comparing two raters (R1 and R2) measuring the US thicknesses of the superficial fascia of the arm and of forearm for the different/levels. Red line = bias or mean difference; dashed lines: 95% limits of agreement (LoAs).

Analysis of the reliability of the US measurements of the thickness of the superficial fascia of the arm and forearm between the two raters (R1 and R2) showed excellent inter-rater reliability for the posterior region of the arm at all levels; in contrast, it showed good inter-rater reliability for the anterior region of the arm at all levels and for all regions/levels of the forearm (Table 11). Bland–Altman plots comparing the two raters for the different topographical regions/levels of deep fascia are shown in Figure 3 and Figure 4. There were no statistical differences between the first session and the second session (*p >* 0.05). The bias, SD of bias, 95% CI, and 95% LoA were, respectively, for the different regions/levels of the arm: Ant 1: −0.1, 0.03, −0.07 to −0.05, −0.12–0.01; Ant 2: −0.1, 0.04, −0.07 to −0.05, −0.15–0.01; Post 1: −0.03, 0.03, −0.04 to −0.03, −0.07–0.02; Post 2: −0.03, 0.03, −0.04 to −0.02, −0.10–0.03. The bias, SD of bias, 95% CI and 95% LoA were, respectively, for the different regions/levels of the forearm: Ant 1: −0.1, 0.04, −0.06 to −0.04, −0.13–0.02; Ant 2: −0.1, 0.04, −0.07 to −0.05, −0.14–0.01; Post 1: −0.1, 0.04, −0.1 to −0.06, −0.14–0.003; Post 2: −0.1, 0.04, −0.07 to −0.05, −0.14–0.01. Figure 12 shows the Bland–Altman plots for the US measurements of the thickness of the deep fascia of the arm between the first session and the second session.

## 4. Discussion

To our current knowledge, this study may be stated as the first study detailing the intra- and inter-reliability of US measurements of fascial thickness in the arm and forearm. The primary aim of this study is to assess the inter-reliability of US measurements of superficial and deep fascia thicknesses in the arm and forearm. Two raters with different backgrounds and levels of expertise assessed the US images and measured the US superficial and deep fascia thicknesses of the arms and forearms of 30 healthy volunteers.

An analysis of our results about the reliability of deep fascia measurements showed excellent inter-rater reliability and intra-rater reliability at different regions/levels. For the anterior region of the arm (Ant 1: ICC_2,__2_ = 0.95; 95% CI = 0.81–0.98; Ant 2: ICC_2,2_ = 0.92; 95% CI = 0.78–0.98) and for the posterior region of the arm (Post 1: ICC_2,2_ = 0.97; 95% CI = 0.87–0.99; Post 2: ICC_2,2_ = 0.93; 95% CI = 0.75–0.97), the inter-reliability was excellent and no significant differences were present between the measurements of the two raters (*p >* 0.05). Similar to the deep fascia of the arm, also for the forearm, the results showed excellent reliability between the two raters for both the anterior region (Ant 1: ICC_2,2_ = 0.98; 95% CI = 0.84–0.99; Ant 2: ICC_2,2_ = 0.97; 95% CI = 0.60–0.99) and the posterior region (Post 1: ICC_2,2_ = 0.97; 95% CI = 0.79–0.99; Post 2: ICC_2,2_ = 0.94; 95% CI = 0.90–0.97). No significant differences were present between the measurements of the two raters (*p >* 0.05).

As to the superficial fascia, the inter-rater reliability showed good and excellent inter-rater reliability for all regions/levels for both the arm and forearm. For the posterior region of the arm (Post 1: ICC_2,2_ = 0.95; 95% CI = 0.70–0.98; Post 2: ICC_2,2_ = 0.94; 95% CI = 0.67–0.98), the inter-reliability was excellent, whereas for the anterior region of the arm (Ant 1: ICC2,2 = 0.85; 95% CI = 0.79–0.88; Ant 2: ICC2,2 = 0.78; 95% CI = 0.80–0.96), the inter-reliability was good. No significant differences were present between the measurements of the two raters (*p >* 0.05). Overall, for the forearm, the results showed good reliability between the two raters for both the anterior region (Ant 1: ICC_2,2_ = 0.86; 95% CI = 0.82–0.96; Ant 2: ICC_2,2_ = 0.83; 95% CI = 0.82–0.95) and the posterior region (Post 1: ICC_2,2_ = 0.86; 95% CI = 0.79–0.96; Post 2: ICC_2,2_ = 0.80; 95% CI = 0.80–0.95). No significant differences were present between the measurements of the two raters (*p >* 0.05).

Bland–Altman plots showed that the limits of agreement were quite narrow and the majority of measurements were contained within the 95% limits of agreement. To interpret these results, the normal distribution of the differences was verified by the Kolmogorov–Smirnov test, confirming the normality of the distribution. Bland–Altman analysis showed moderate and optimal agreement between the two raters in the assessment of deep and superficial fascia in the different regions/levels of the arm and forearm. The biases were low for both anterior and posterior regions/levels of deep fascia of the arm and forearm. As to the superficial fascia, the biases were low for both regions but slightly higher than those observed for the deep fascia of the arm. Moreover, overall, for the forearm, the biases were low for both regions but slightly higher than those observed for the deep fascia of the arm.

The regions/levels of the forearm and arm that have good reliability and moderate agreement are points in which the superficial fascia adheres more firmly to the deep fascia; therefore, thicker retinacula cutis can confuse the US images. Moreover, the results confirmed, as has been demonstrated by other studies, that in the evaluation of superficial fascia, the inter-reliability is not as excellent as for the deep fascia; however, it maintains good reliability. Furthermore, the Bland–Altman analysis showed that as most measurements fall below the zero line for superficial fascia, R2 consistently measured a higher thickness than R1, indicating that slight disagreement is more systematic than random, underlining how important continuous training is to acquire specific knowledge for the recognition of these anatomical structures. This aspect underlines that the measurement of the superficial fascia, in particular in the forearm, is more difficult as it is a smaller structure than the deep fascia [30], although there are no significant differences between the measurements of the two raters.

These data confirm that US imaging is an excellent tool to evaluate the fasciae, both superficial and deep, and that excellent inter-rater reliability exists. The reliability found in this study is in line with the results of other reliability studies that have evaluated other areas, such as abdominal fasciae [23] and lower limb fasciae [17,21].

In light of these results, the excellent and good inter-reliability values can be explained by a single rater taking the US images using the same protocol. The possible error by image acquisition was eliminated as a source of variation. US measurements were made on the recorded images by the two raters using the same protocol to assess US images and measure the thicknesses.

The choice to have the US images taken by R1 was made to avoid inter-rater differences that could be linked to the tilting of the transducer or the placement of pressure or the imperfect use of the protocol by R2 as the procedure takes experience and practice.

As reported by other studies, inter-rater reliability is defined as the degree of agreement among measurements by multiple examiners, and it is considered a key factor in research, guaranteeing that different researchers using the same procedure/protocol obtain the same results. In daily practice, in the clinical environment, inter-rater reliability enables physicians to diagnose and monitor a patient’s progress reliably over time [31,32].

Therefore, the first step in the training path of the novice R2 is to acquire the specific knowledge of recognizing the anatomical structures through the best ultrasound images; then, with time, experience, and, above all, the study of the various protocols [14,15,17,21,23], he will arrive at the final step of taking the images and their analysis. The high variability of these structures imposes specific steps to acquiring the skills, from anatomy to sono-anatomy.

This is the first work, to our knowledge, to examine the intra- and inter-rater reliability in US measurements of superficial and deep fasciae thicknesses in the arm and forearm, as assessed by two raters with different knowledge and experience. The agreement between R1 and R2 suggests and supports that the initial training to increase fascial anatomical knowledge and translate it to sono-anatomy is essential for the good and excellent interpretation and analysis of US images. Moreover, it underlines that it is fundamental to take the images in an optimal manner, arriving at this step of training after having obtained optimal knowledge and experience in skeletal–muscle US imaging.

### Limitations of the Study

This study was intentionally focused on a healthy cohort to demonstrate the importance of continuous training to acquire specific knowledge for the recognition of anatomical structures. Nevertheless, despite rigorous efforts (multiple controls of the measurement region/level by each rater) and previous training, the level of experience of the raters, especially in such examiner-dependent assessments as US imaging, must be considered. Finally, the small number of healthy volunteers and the importance of the qualitative aspects in the fascial assessment mean that it is not possible to statistically analyze the prevalence of US findings and to explain their possible causes, prognostic significance, and therapeutic implications.

## 5. Conclusions

The study demonstrates that US measurements of superficial fascia and deep fascia thicknesses in the arm and forearm can be considered reliable. Knowledge of the anatomy and sono-anatomy of fasciae and expertise in the analysis of US fasciae images are essential for reliable results. After a complete and detailed first step of training, a novice rater can also perform measurements of US fascial images with good and excellent reliability. The last step will be to acquire the knowledge of protocols after a period of training.

## Figures and Tables

**Table 1:** Descriptive data of the sample.

Descriptive Statistics	Age (years)	BMI (kg/m^2^)	Height (cm)	Weight (kg)
Number of values	30	30	30	30
Minimum	20	15.79	158	43
Maximum	60	31.6	183	87
Range	40	15.81	25	44
Mean	33.23	23.27	170.7	67.28
Standard deviation	13.31	3.692	6.865	13.54
Coefficient of variation	40.06%	15.86%	4.022%	20.12%

BMI = Body Mass Index.

**Table 2:** Intra-rater reliability of the ultrasound measurements within different regions/levels of the deep fascia of the arm between two sessions by R1.

Region	Level	ICC_3,2_	95% C.I.
Anterior	Ant 1	0.97	0.91–0.98
Anterior	Ant 2	0.97	0.84–0.98
Posterior	Post 1	0.99	0.97–0.99
Posterior	Post 2	0.97	0.87–0.99

ICC_3,2_ = intra-class correlation coefficient type 3,2; C.I. = confidence interval.

**Table 3:** Intra-rater reliability of the ultrasound measurements within different regions/levels of the deep fascia of the arm between two sessions by R2.

Region	Level	ICC_3,2_	95% C.I.
Anterior	Ant 1	0.94	0.53–0.98
Anterior	Ant 2	0.94	0.69–0.98
Posterior	Post 1	0.95	0.71–0.98
Posterior	Post 2	0.96	0.77–0.98

**Table 4:** Intra-rater reliability of the ultrasound measurements within different regions/levels of the deep fascia of the forearm between two sessions by R1.

Region	Level	ICC_3,2_	95% C.I.
Anterior	Ant 1	0.96	0.90–0.98
Anterior	Ant 2	0.95	0.80–0.98
Posterior	Post 1	0.95	0.65–0.98
Posterior	Post 2	0.97	0.93–0.98

**Table 5:** Intra-rater reliability of the ultrasound measurements within different regions/levels of the deep fascia of the forearm between two sessions by R2.

Region	Level	ICC_3,2_	95% C.I.
Anterior	Ant 1	0.97	0.86–0.99
Anterior	Ant 2	0.96	0.62–0.98
Posterior	Post 1	0.94	0.67–0.98
Posterior	Post 2	0.95	0.67–0.98

**Table 6:** Intra-rater reliability of the ultrasound measurements within different regions/levels of the superficial fascia of the arm between two sessions by R1.

Region	Level	ICC_3,2_	95% C.I.
Anterior	Ant 1	0.96	0.91–0.98
Anterior	Ant 2	0.96	0.90–0.98
Posterior	Post 1	0.96	0.90–0.98
Posterior	Post 2	0.96	0.84–0.98

**Table 7:** Intra-rater reliability of the ultrasound measurements within different regions/levels of the superficial fascia of the arm between two sessions by R2.

Region	Level	ICC_3,2_	95% C.I.
Anterior	Ant 1	0.96	0.87–0.98
Anterior	Ant 2	0.93	0.78–0.97
Posterior	Post 1	0.94	0.84–0.97
Posterior	Post 2	0.95	0.82–0.98

**Table 8:** Intra-rater reliability of the ultrasound measurements within different regions/levels of the superficial fascia of the forearm between two sessions by R1.

Region	Level	ICC_3,2_	95% C.I.
Anterior	Ant 1	0.90	0.75–0.95
Anterior	Ant 2	0.89	0.74–0.95
Posterior	Post 1	0.96	0.89–0.98
Posterior	Post 2	0.91	0.70–0.96

**Table 9:** Intra-rater reliability of the ultrasound measurements within different regions/levels of the superficial fascia of the forearm between two sessions by R2.

Region	Level	ICC_3,2_	95% C.I.
Anterior	Ant 1	0.90	0.76–0.95
Anterior	Ant 2	0.78	0.55–0.89
Posterior	Post 1	0.92	0.84–0.96
Posterior	Post 2	0.89	0.57–0.95

**Table 10:** Inter-rater reliability of the ultrasound measurements within different regions/levels of the deep fascia of the arm and forearm between the two raters.

Anatomical District	Region	Level	ICC_2,2_	95% C.I.
Arm	Anterior	Ant 1	0.95	0.81–0.98
Arm	Anterior	Ant 2	0.92	0.78–0.98
Arm	Posterior	Post 1	0.97	0.87–0.99
Arm	Posterior	Post 2	0.93	0.75–0.97
Forearm	Anterior	Ant 1	0.98	0.84–0.99
Forearm	Anterior	Ant 2	0.97	0.60–0.99
Forearm	Posterior	Post 1	0.97	0.79–0.99
Forearm	Posterior	Post 2	0.94	0.90–0.97

**Table 11:** Inter-rater reliability of the ultrasound measurements within different regions/levels of the superficial fascia of the arm and forearm between the two raters.

Anatomical District	Region	Level	ICC_2,2_	95% C.I.
Arm	Anterior	Ant 1	0.85	0.79–0.88
Arm	Anterior	Ant 2	0.78	0.80–0.96
Arm	Posterior	Post 1	0.95	0.70–0.98
Arm	Posterior	Post 2	0.94	0.67–0.98
Forearm	Anterior	Ant 1	0.86	0.82–0.96
Forearm	Anterior	Ant 2	0.83	0.82–0.95
Forearm	Posterior	Post 1	0.86	0.79–0.96
Forearm	Posterior	Post 2	0.82	0.80–0.95

## Data Availability

The data presented in this study are available upon request from the corresponding author. The data are not publicly available due to privacy.
